# Visualization of Light-Impinging Geometry in Nonlinear Photocurrents of Vertical Optoelectronic Devices

**DOI:** 10.3390/ma18153503

**Published:** 2025-07-25

**Authors:** Hacer Koc, Jianbin Chen, Dawei Gu, Mustafa Eginligil

**Affiliations:** 1 Key Laboratory of Flexible Electronics (KLoFE), Institute of Advanced Materials (IAM), School of Flexible Electronics (Future Technologies), Nanjing Tech University, 30 South Puzhu Road, Nanjing 211816, China; haccerkoc@gmail.com (H.K.); 18258857615@163.com (J.C.); 2 Department of Physics, School of Physical and Mathematical Sciences, Nanjing Tech University, Nanjing 210009, China

**Keywords:** nonlinear photocurrent, vertical device structures, light–matter interaction

## Abstract

Nonlinear photocurrents (NPs) are electrical currents expected to be measured at the electrodes of a device consisting of an active area, sensitive to light, with a higher-order in-electric field where light-impinging geometry (LIG) is the determining factor in the experimental observation. Although the phenomenology of this light–matter interaction is clear for light directed on a lateral device plane with well-defined azimuthal and incidence angles, as well as light polarization angle, it can be quite complicated for a vertical device structure and reconsideration of the expected NP contributions is necessary in the latter case. In this study, we used a visual approach to describe the LIG for vertical device structures using a specific example of a photodiode, and showed that these angles must be redefined, namely, the interchangeability of azimuthal and incidence angles. The influence of device geometry-dependent optical illumination is reflected on the behavior of NP; therefore, the NPs that are known to be forbidden in certain LIGs can be allowed and vice versa. These results pave the way for the utilization of NPs in flexible optoelectronic applications.

## 1. Introduction

The polarization state of a light field (or the electric field of the electromagnetic wave for a nonmagnetic medium) can be varied using optical instruments, e.g., wave retarders; then, light with a certain polarization state can impinge on the surface of a medium and regulate its polarization. The interaction of light with the medium can be linear or nonlinear depending on the electric field’s first- or higher-order expansion and microscopic polarizability (see Section 3.1 for an explanation) [1]. As a result of this interaction, nonlinear photocurrents (NPs) can be observed at second and higher orders depending on the symmetry of the medium. In particular, second-order NPs have been an important point of interest in fundamental studies of bulk materials [2,3,4,5,6,7,8,9]. The crystal symmetry of the media, the asymmetric distribution of charge carriers’ momenta, and the asymmetric scattering of free carriers have been found to be crucial in determining the nature of NPs; recently, these properties have attracted a lot of attention in low-dimensional materials [10,11,12,13,14].

The importance of light-impinging geometry (LIG)—the way light approaches the surface of a medium, including the direction and the polarization state—has been emphasized in several works, such as in graphene, the prototype low-dimensional material for NPs [11,15]. In other cases of 2D transition metal dichalcogenides (TMDs) [16,17,18,19,20,21] and relatively thicker materials like topological insulators [22,23,24], LIG was considered in lateral device structures with Landauer-type configurations in which NP signal collection electrodes are in the sample plane. On the other hand, in materials with special geometric structures such as edge-embedded TMD heterostructures [25], although the device structure is lateral, LIG can lead to unprecedented and enhanced NPs. This is due to the fact that the NP expected from lateral device geometry, as in [16,17,18,19,20,21], is transformed to a sophisticated one, with symmetry-reduction-based NP enhancement, as expected in graphene NPs [11]. Similarly, the NPs directly observed in single-crystal [26] and polycrystalline [27] hybrid organic–inorganic perovskites (HOIPs) in lateral device geometries can take various forms in terms of different internal geometries, such as multiple quantum wells of HOIPs [28], aqueously synthesized HOIPs [29], and stepwise composition gradient epitaxially grown hybrid perovskites [30], as well as layered perovskites [13]. Therefore, for any device geometry, LIG has to be well defined and understood for extraction and for distinguishing NP contributions.

In low-dimensional structures, more specifically in 2D material heterostructures, it was predicted that the linear photocurrent of vertical device structures (where the electrodes are at the top and bottom of the channel) would be enhanced under light irradiation [31] if the central region of the device is irradiated. This expectation is also applicable for NPs in 2D materials, where most of the measurements so far have been performed in lateral device geometries [11,15]. Furthermore, in organic solar cells, for light propagating normal to the device plane and where electrodes were placed on the top and bottom of the device, alternating the polarization state of light from linearly to circularly polarized light yielded a linear photocurrent, which could not be observed in inorganic counterparts [32]. In similar devices but with an HOIP as the active layer, which acquires strong spin–orbit coupling, this switching of light polarization from linear to circular manifested spin effects on photovoltaic actions [33]. However, the influence of this LIG on nonlinear photocurrents in vertical devices, particularly the aforementioned HOIP, is still not clear. For instance, it would be quite informative to identify the NP contributions in the case of vertical devices with a chiral 2D HOIP as the active layer [34], as well as multiple quantum wells [28] and layered perovskites [13].

In this work, the critical role of device and light geometries in non-centrosymmetric media [35], which has not been considered in depth so far, was rigorously studied from a visual approach. Starting from the well-known phenomenology, the LIG was visualized in vertical devices, and the expected NP contributions with specific angle dependencies were identified. A typical vertical heterostructure of an HOIP was utilized in this visualization process without the loss of generality, and the photocurrent was analyzed accordingly. As a result, it was found that some photocurrent contributions that are not expected to be allowed in certain LIGs can actually exist, and vice versa. These results suggest that the NPs in vertical device structures have to be treated quite differently than the typical lateral ones and there is a high potential of NP applications in flexible optoelectronics.

## 2. Materials and Methods

**Figure 1:**
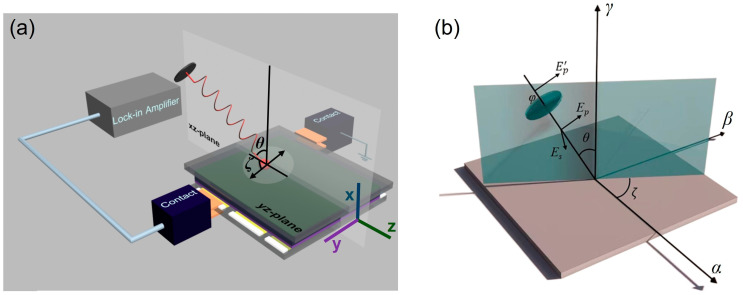
(**a**) The nonlinear photocurrent setup for a vertical photodiode. Here, in this specific representation, the light is impinging on the yz-plane of the device along the xz-plane. (**b**) The light-impinging geometry for a typical lateral device, with generalized αβγ Cartesian coordinate system.

The nonlinear photocurrent experimental setup is depicted in Figure 1a. The primary measurement technique involves acquiring a light-polarization-dependent nonlinear photocurrent (second order) using a lock-in amplifier—a well-known standard approach. This setup resembles a linear photocurrent setup in terms of laser excitation with an energy of 1.96 eV to excite the device surface. The laser power used for an HOIP-based vertical device was typically 5 μW with a half of a mm beam spot and operated in the linear dependence regime of laser power (no heating effects). A chopper was utilized to frequency-lock the light signal, which was then phase-sensitively detected by the lock-in amplifier (placed between the laser source and the wave retarder not included in the figure for simplicity). Initially, the light was p-polarized, followed by the use of a quarter-wave plate (QWP or λ/4) as a retarder to achieve elliptical polarization. The fast axis was set at 0° as the angle of photon polarization (φ), which could be adjusted by rotating the QWP from 0° to 360°. Subsequently, a mirror and a focusing lens unit (not included in figures for simplicity) were employed to concentrate the light onto the sample plane at an angle of incidence θ and azimuthal angle ζ, as defined from the +x axis and +y axis, respectively, in this particular case. A typical scan from 0° to 360° of φ by rotating QWP was carried out in steps of 5° and averaged out of multiple scans. In this paper, typical samples of HOIP-based vertical photodiodes will be presented. More specifically, they consist of an active layer of HOIP sandwiched between an electron transport layer and a hole transport layer. As shown in Figure 1a, only one specific contact electrode (top electrode in this case) and a laser beam point were used until the completion of the measurement.

The visualization of light impinging geometries on vertical device structures was performed by AutoCAD 3ds Max 2019. The actual sets of vertical devices were photodiodes based on HOIP active layers (with a Au electrode, lateral device size of 1.5 × 2 cm, sandwiched between transport layers) and prepared according to previous reports [36,37].

## 3. Results

The NP measurement results obtained using the experimental setup presented in Figure 1a can only be understood after a proper visualization of light-impinging geometries on vertical device structures. But before that, the phenomenology of NPs will be introduced in Section 3.1 to emphasize the importance of proper visualization in the evaluation of NP contributions, which is later given in detail in Section 3.2, as the main message. Finally, the expected NP contributions based on visualization are confirmed by the experiments in Section 3.3.

### 3.1. Phenomenology

The general description of the polarized light–media interaction and the angles mentioned in the previous section are described in Figure 1b. This is the standard phenomenology, where one can perceive a resulting photocurrent $j_{\alpha }\left(r,t\right)$ upon the action of the electric field E(ω,q) due to light on the sample plane with αβγ-coordinate system. The general form of these critical angles determining the polarization-dependent photocurrent $j_{\alpha }\left(r,t\right)$ is as follows: the angle determined from the initial linear polarization of E(ω,q) or so-called angle of photon polarization (φ); the angle determined from the normal to the surface (the γ-direction) to the direction of propagation of $E\left(\omega ,q\right)$, known as the angle of incidence (θ); and the angle determined, on the surface, from the α-direction to the β-direction, labeled as the azimuthal angle (ζ). As an example, the light was first p-polarized using a polarizer. In this case, E(ω,q) can be simply considered as $E_{p}^{\prime }$ and is incident on a QWP. After that, by rotating the QWP from its fast axis, where φ=0°, it is possible to change the p-polarization of E(ω,q) to elliptical, linear s-polarization, and circular polarizations. Upon leaving the QWP, E(ω,q) can have components in the $E_{s}$ and $E_{p}$ directions. In this situation, the polarization is linear for φ=nπ/2 or circular for φ=(2n+1)π/4 for n=0, ±1±2±3,±4, and so on. Thus, the polarized E(ω,q) will impinge on the αβ-plane of the sample with a certain θ, leading to a polarization-dependent photocurrent $j_{\alpha }\left(r,t\right)$ for an azimuthal angle of ζ.

The polarization vector ***P*** and the polarizability has a relationship via the electric field ***E***, which has the form

(1) $$E\left(r,t\right)=E\left(\omega ,q\right)e^{i\left(qr-\omega t\right)}+E^{*}(\omega ,q)e^{-i\left(qr-\omega t\right)}$$

where ***r*** and *t* are the positions of the plane wave and ***q*** and *ω* are the wave vector and frequency of the light. The relationship between the polarizability tensor χ and the polarization vector ***P*** can be simply given as

(2) $$P={\;\varepsilon }_{0}\;(\chi^{\left(1\right)}E+\chi^{\left(2\right)}\;EE+\ldots )$$

where $\varepsilon_{0}$ is the vacuum permittivity and χ^(n)^ is the nth order polarizability tensor, for *n* = 1, 2, and so on (only shown up to second order). The general form of $E_{p}^{\prime }$ shown in Figure 1b is E(ω,q) and can take the form of a plane wave for its interaction with a sample to lead to a photocurrent, which is the product of conductivity tensor σ, and E(ω,q). Here, specifically, it is given as second order:

(3) $$j_{\alpha }\left(r,t\right)=\left[\sigma_{\alpha \beta }^{\left(1\right)}E_{\beta }\left(\omega ,q\right)e^{i\left(qr-\omega t\right)}\right]+\left[\sigma_{\alpha \beta \gamma }^{\left(2\right)}E_{\beta }\left(\omega ,q\right)E_{\gamma }^{*}\left(\omega ,q\right)\right]$$

Equation (3) expresses the second-order expansion of the photocurrent and α, β, and γ are the subscript corresponding to previously defined Cartesian coordinate system. The first term described by $\sigma_{\alpha \beta }^{\left(1\right)}$ is the linear photocurrent. The following term is the second-order nonlinear photocurrent, where the third rank conductivity tensors $\sigma_{\alpha \beta \gamma }^{\left(2\right)}$ are associated with the DC current, which is the only nonzero current that can be obtained to second order and that was measured as a nonlinear photocurrent in these experiments.

### 3.2. Light-Impinging Geometries on a Vertical Device Structure

**Figure 2:**
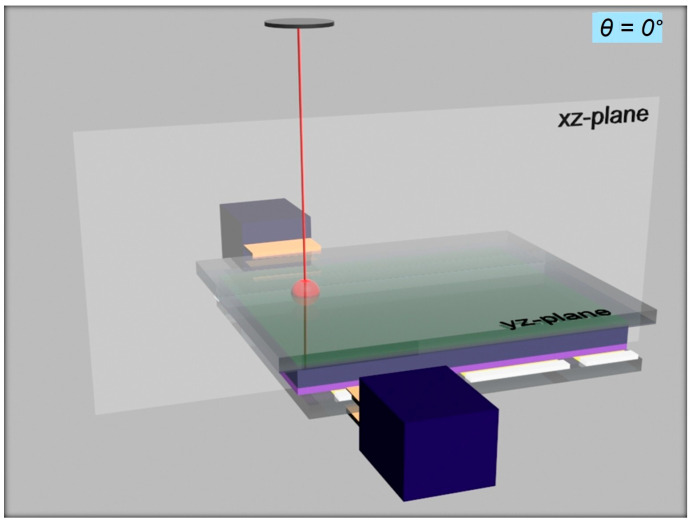
Initial state of light impinging onto a vertical device surface, where the nominal angle of incidence θ = 90°, at the intersection of the xz-plane and the xy-plane to be varied along the xz-plane.

Considering the expression for the NP on the right-hand side of Equation (3), various LIGs can be described. The angles shown in Figure 1b for a typical lateral device geometry make a transformation to the configuration shown in Figure 1a, which is not straightforward to understand on first look. However, the previously defined coordinate system in Figure 1a is helpful. In a lateral device, the sample plane is usually defined as the xy-plane, where the electrodes are located within with a channel length, and the z-direction is the one normal to the sample plane and the electrodes. However, in this vertical device structure, taking the standard description of Figure 1b, the angle of incidence θ, between the normal (which is the *x*-axis in this case) and the direction for which ζ = 90° was set, can be seen in Figure 2. Starting from the given position, the laser line will be within the xz-plane and can be varied along this plane with increasing values of θ.

**Figure 3:**
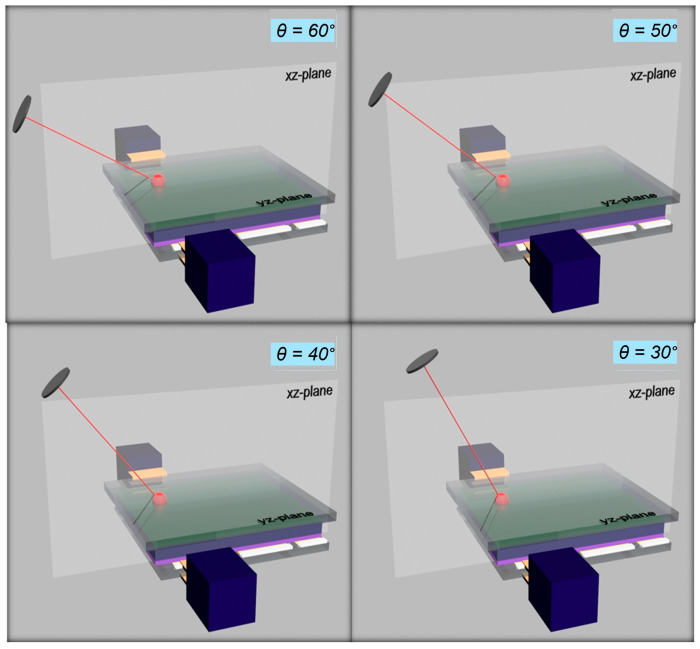
Snapshots of 3D simulation for varying nominal angle of incidence θ at fixed azimuthal angle of ζ = 90° incidence θ, for light impinging onto a vertical device structure.

Given the fact that the electrodes in Figure 2 are at the top and bottom of the device and the active layer is in the yz-plane, varying the nominal angle of incidence θ within the xz-plane would mean the excited carriers would flow towards one specific electrode and the direction of the flow could change sign if the light is incident at angles θ > 90°. In this respect, the angle of incidence θ in a vertical device of Figure 2 acts like the azimuthal angle of the case of a lateral device of Figure 1b; therefore, the nominal θ is an azimuthal angle in reality. The variation in nominal θ between 30 and 60° in the case of a vertical device is displayed in Figure 3, in steps of 10°, for visualization purposes. In principle, this angle could be further changed, but for θ > 60°, light would be largely dispersed and beam shifts could occur.

**Figure 4:**
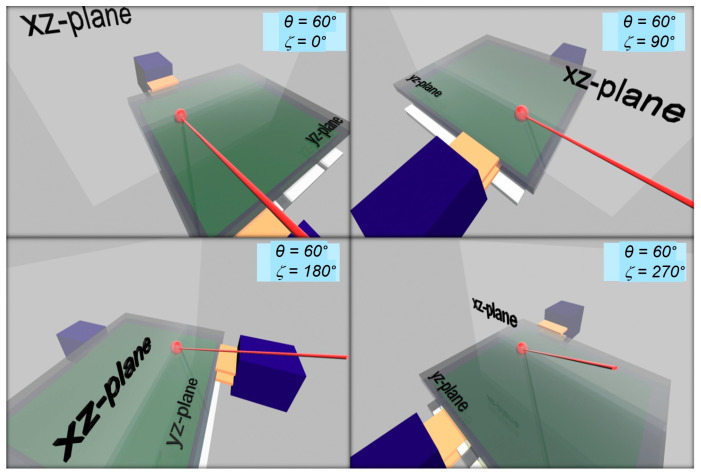
Snapshots of 3D simulation for varying nominal azimuthal angle and fixed angle of incidence θ for light impinging onto a vertical device structure.

In the case of fixing the nominal angle of incidence θ at a typical oblique incidence of 60° and varying the nominal azimuthal angle ζ, the situation will be like that in Figure 4 (see Supplementary Video S1 for a better view). Here, snapshots of the LIG for ζ = 0°, 90°, 180°, and 270° are presented. Initially, for θ = 60° and ζ = 0°, the laser beam is in the xy-plane and perpendicular to the xz-plane. This is the situation where the photo-excited carriers are expected to move towards the top electrode. Next, for θ = 60° and ζ = 90°, the laser beam is in the xz-plane and perpendicular to the xy-plane, which makes the photo-excited carriers move to either electrode and the state of light polarization coupling with media determines the direction. Then, for θ = 60° and ζ = 180°, the laser beam is back in the xy-plane and perpendicular to the xz-plane. This is the situation where the photo-excited carriers are expected to move toward the bottom electrode. Finally, for θ = 60° and ζ = 270°, the laser beam is again in the xz-plane and perpendicular to the xy-plane; this the same situation as the case of θ = 60° and ζ = 90°.

### 3.3. Nonlinear Photocurrent in an HOIP-Based Vertical Device

**Figure 5:**
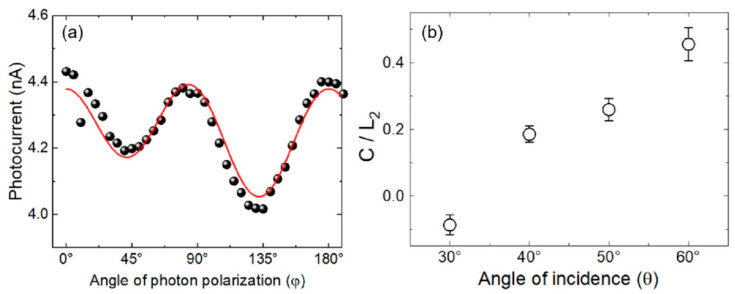
(**a**) Nonlinear photocurrent (NP) as a function of angle of photon polarization φ for HOIP-based vertical photodiode for a fixed angle of incidence θ = 60° and ζ = 180°. The fitting curve is the phenomenological photocurrent formula from which NP contributions C, $L_{1}$, and $L_{2}$ can be extracted. (**b**) The figure of merit for NP, C/$L_{2}$ is plotted using the values determined from fittings at a fixed azimuthal angle of ζ = 180°.

The LIGs described in the previous section are applicable to any vertical device structures. Here, the nonlinear photocurrent behavior of HOIP active layer-based samples will be given as an example. Figure 5a shows the photocurrent as a function of the angle of photon polarization φ obtained by varying the angle of QWP for a fixed angle of incidence θ = 60° and ζ = 180°. The data points were fit to the phenomenological photocurrent formula

(4) $$j_{\alpha }=C\sin\left(2\varphi \right)+L_{1}\sin\left(4\varphi \right)+L_{2}\cos\left(4\varphi \right)+D$$

where C is the nonlinear photocurrent contribution accounting for light excitation with a circularly polarized state, while $L_{1}$ and $L_{2}$ are nonlinear photocurrent contributions responsible for linearly polarized excitation and D is the polarization-independent contribution. Equation (4) is the outcome of the second term on the right-hand side of Equation (3) after following a series of operations [38] using the Jones calculus [39].

It is apparent that in Figure 5a, there are two dips in the fitting curve at ~45° and 135° and the variation between their corresponding photocurrent values exhibits a typical influence of $\sin\left(2\varphi \right)$ and *C* term. Among the linear terms $L_{1}$ is relatively weaker than $L_{2}$ and a figure of merit can be defined as the effect of circularly polarized excitation with respect to the linearly polarized excitation, as C/$L_{2}$. This ratio was determined in a way similar to the procedure of Figure 5a for three more fixed angles of incidence θ at ζ = 180° (Supplementary Materials Figures S1–S3) and plotted in Figure 5b. All of the components were extracted and are tabulated in Supplementary Materials Table S1. The dependence on θ shows a monotonic increase, and the strongest C/$L_{2}$ ratio can be seen at larger θ = 60°. The photocurrents for θ < 30° and θ > 60° at fixed ζ = 180° exhibited large fluctuations in angle of photon polarization φ dependence and it was not possible to extract NP terms from the fitting of the data to Equation (4). This behavior for θ < 30° and θ > 60° can be simply ascribed to a large variation in refraction at the interface between the HOIP-active layer and the other layers, and the large spreading of the incident beam at the surface, respectively, to be discussed later. However, the observed behavior between 30° < θ < 60° is reminiscent of the azimuthal angle dependence of the photocurrent in lateral device geometry, in which the C and C/$L_{2}$ ratio becomes gradually larger starting from ζ = 0° and reaching a maximum of ζ = 90° [15,16,17,18,19,20,21,22,23,24,26,27,28].

**Figure 6:**
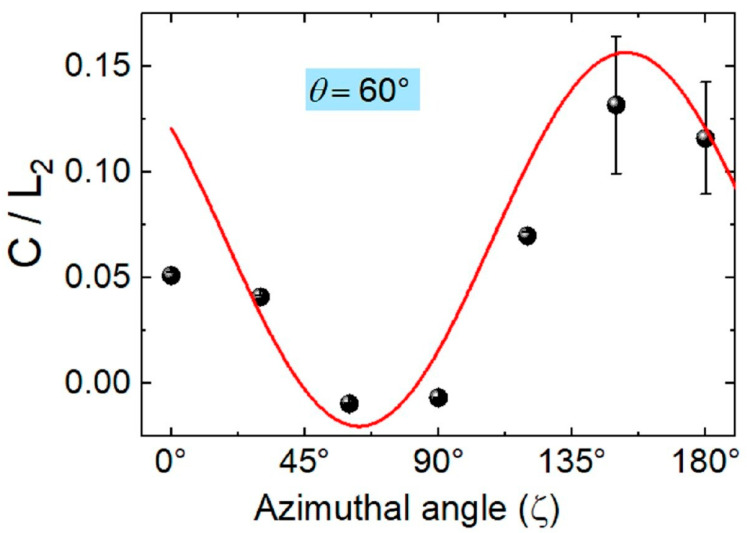
The figure of merit for nonlinear photocurrents, the ratio of C/$L_{2}$, for an HOIP-based vertical photodiode is plotted using the values determined from fittings at fixed angle of incidence θ = 60° as a function of the azimuthal angle of ζ. The fitting curve is a sinusoidal function.

The above observation also manifested itself in azimuthal angle ζ dependence at fixed θ = 60°, as seen in Figure 6, in a similar device. Again, the C/$L_{2}$ ratio, which was determined in a way similar to the procedure of Figure 5a for seven different fixed azimuthal angles ζ, are plotted in Figure 6. The ratio is negative and has values almost zero around ζ = 90° and has higher positive values around ζ = 0° and ζ = 180°. This is quite similar to the case of the lateral geometry devices of Figure 1b, where θ = 0° yields the lowest or vanishing values of C/$L_{2}$ ratio, and then increases sinusoidally with increasing θ. However, in lateral devices, it is not possible to measure the photocurrent when approaching θ = 90° and above. The shift in the sinusoidal fitting curve in Figure 6 accounts for the difference in the influence of the photo-excited carrier flow (towards the bottom electrode or the top electrode) upon the change in light propagation direction.

## 4. Discussion

The nonlinear photocurrent (NP) data and the analysis provided in the previous section clearly demonstrated that LIGs can be critical in the determination of NP contributions. It is essential to obtain stable NP data to determine reliable NP contributions. The simple phenomenology in a lateral device becomes complicated in vertical devices like photodiodes due to the interfaces that light passes through. The refraction can vary at large angles. On the other hand, the polarized light beam can be sensitive at small angles. In either case, unstable NPs can be obtained and it is not possible to extract NP contributions to make any evaluation. Additionally, the process of varying the light polarization via a QWP may also lead to beam shifts [40] that could affect the NP in general.

While these unwanted effects naturally emerge due to the reasons mentioned above, they neither completely mask the main expected phenomenology nor hinder the NP contributions in vertical device structures. On the contrary, the obtained results here, including the missing NPs in small and large angles of incidence, underline the potential of covering the specific details of NP contributions, such as $C_{1}$ and $C_{2}$ components of *C*, in which $C_{2}$ is usually underestimated in lateral device structures. The NP contribution $C_{2}$ is usually referred to as inhomogeneity [28], which may become important at the onset of large NP fluctuations for the specific case mentioned here and is normally taken to be negligible and not mentioned in typical analysis. This requires further attention and has to be studied systematically. In addition, the appearance of NP contributions within the narrow window of angle of incidence, the interchanged behavior of the azimuthal angle with the angle of incidence, and experimental realization of the expected outcome of the LIG visualization suggest that the physical mechanisms behind these NP contributions are quite robust, requiring in-depth analysis.

On a further note, there is an obvious intriguing relationship between the device geometry and the LIG, which was partially addressed in this work and may be applicable to other flexible optoelectronic devices that usually deal with devices on flexible substrates. To be specific, for example, the origin of the C term may be due to the photogalvanic effects [2,3,4,5,6,7,8,9] or photon drag effects [41,42], depending on the symmetry of light-impinging media and it is an electric current obtained upon excitation by light helicity. The circular photogalvanic effect (which is known as a spin-dependent term and is becoming important for materials like HOIPs that possess strong spin–orbit coupling [26]) is allowed in reduced symmetries, while the circular photon drag effect is not allowed at normal incidence. Therefore, the NP contribution of the C term, that is known to be forbidden in normal incidence in lateral devices, can be allowed in vertical devices; however, it may not be allowed for certain azimuthal angles. Moreover, the circular photogalvanic effect that is not expected to be seen in certain azimuthal angles can now be the dominating NP contribution. A combination of two devices types, lateral and vertical, may initiate a new platform to implement NPs and the functionality mentioned here could provide another dimension for flexible optoelectronic devices.

## 5. Conclusions

In summary, for nonlinear photocurrents, light-impinging geometry (LIG) and device geometries are two important determining factors in which the phenomenology of the lateral device is straightforward, with well-defined azimuthal and incidence angles and light polarization angles, but this not that simple in a vertical device structure. In this work, various LIGs for vertical device structures were visualized and the expectation of interchangeability between the angle of incidence and the azimuthal angle was evidenced by nonlinear photocurrent measurements in HOIP active layer-based vertical photodiodes. This observation exemplified the unprecedented power of nonlinear photocurrents to be used in various device configurations and showed that the nonlinear photocurrent response can have a different nature in various LIGs for different device geometries. Future work should be directed toward the combination of lateral and vertical device structures to enhance the capabilities of nonlinear photocurrents and the implementation of various nonlinear photocurrent contributions in flexible optoelectronics.

## Data Availability

The original contributions presented in the study are included in the article/Supplementary Materials. Further inquiries can be directed to the corresponding authors.

## Supplementary Materials

The following supporting information can be downloaded at https://www.mdpi.com/article/10.3390/ma18153503/s1: Figure S1: Angle of photon polarization dependence of photocurrent at an angle of incidence θ = 30° and at a fixed azimuthal angle of ζ = 180°; Figure S2: Angle of photon polarization dependence of photocurrent at an angle of incidence θ = 40° and at a fixed azimuthal angle of ζ = 180°; Figure S3: Angle of photon polarization dependence of photocurrent at an angle of incidence θ = 50° and at a fixed azimuthal angle of ζ = 180°; Table S1: Extracted photocurrent contributions from the phenomenological photocurrent formula (Equation (4) in main text) fitting for various angle of incidence θ at a fixed azimuthal angle of ζ = 180°; Video S1: Visualization of light impinging on the sample plane with a typical nominal oblique incidence of 60° and varying the nominal azimuthal angle ζ.
