# Supplementary material for: Visualization of Light-Impinging Geometry in Nonlinear Photocurrents of Vertical Optoelectronic Devices
Source: Materials (Basel). 2025 Jul 25;18(15):3503. doi: 10.3390/ma18153503 (PMC12347641; doi:10.3390/ma18153503)
Supplement: Supplementary file 1 [file materials-18-03503-s001.zip › MDPI Materials_Visualization of light impinging geometry - Supp Inf.pdf]

Supplementary Information for

Visualization of light impinging geometry in nonlinear photocurrents of vertical optoelectronic devices

Hacer Koc [1], Jianbin Chen [1], Dawei Gu [2,*] and Mustafa Eginligil [1,*]

[1] Key Laboratory of Flexible Electronics (KLoFE) & Institute of Advanced Materials (IAM), School of Flexible Electronics (Future Technologies), Nanjing Tech University, 30 South Puzhu Road, Nanjing 211816, China

[2] Department of Physics, School of Physical and Mathematical Sciences, Nanjing Tech University, Nanjing 210009, China

*Correspondence: M.E.: iameginligil@njtech.edu.cn; D.G.: dwgu@njtech.edu.cn

This file includes 3 figures and 1 table.

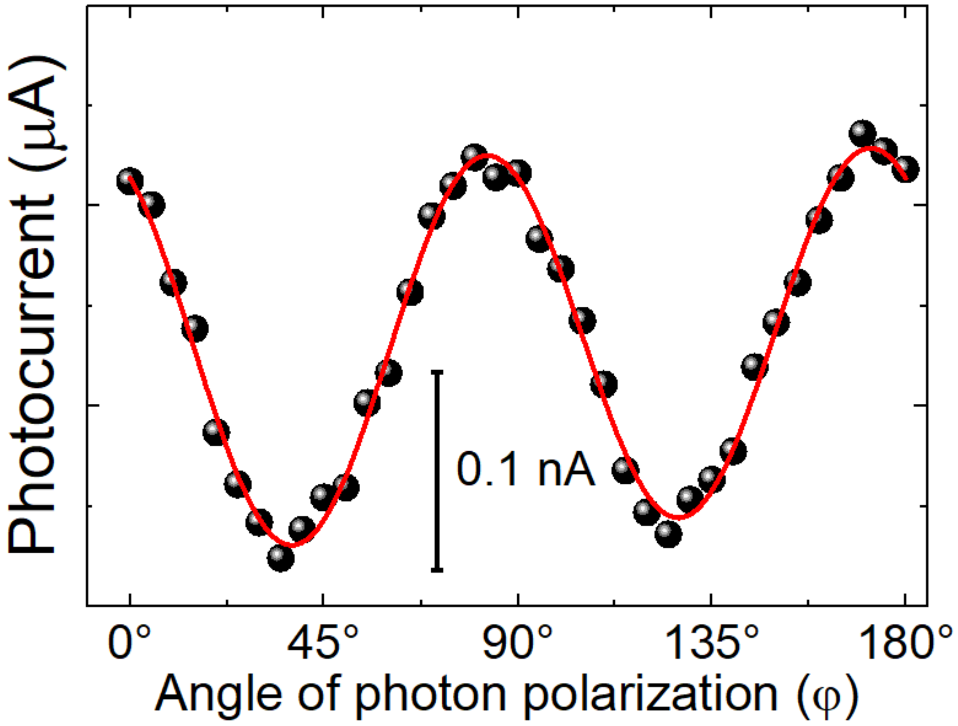

Figure S1 Angle of photon polarization dependence of photocurrent at an angle of incidence θ = 30° and at a fixed azimuthal angle of ζ = 180°.

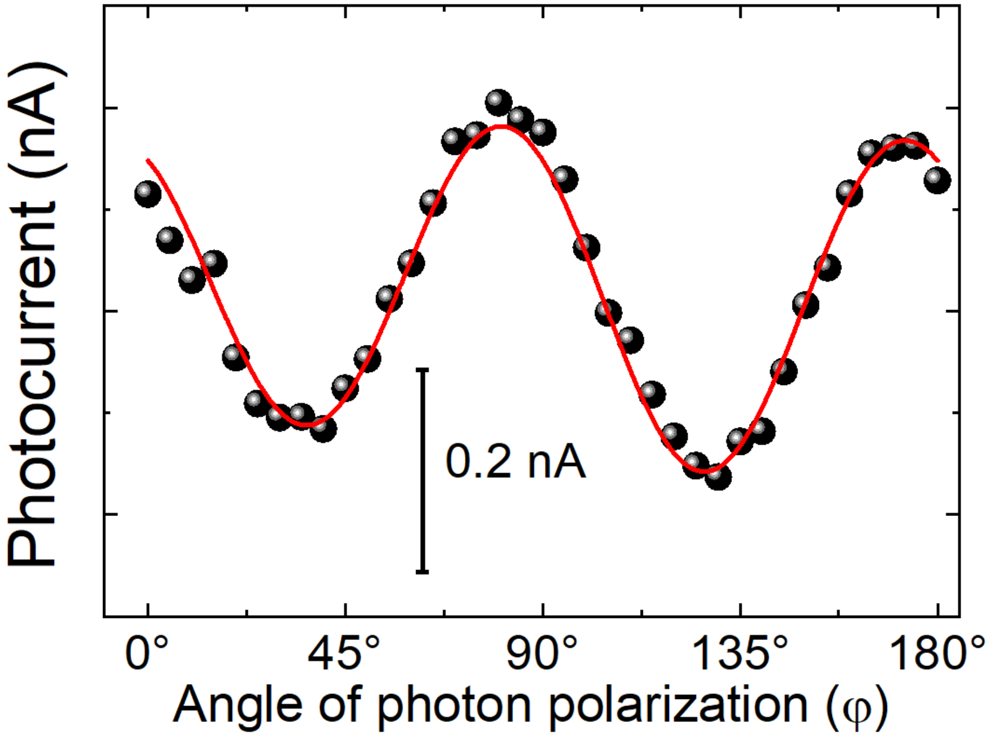

Figure S2 Angle of photon polarization dependence of photocurrent at an angle of incidence θ = 40° and at a fixed azimuthal angle of ζ = 180°.

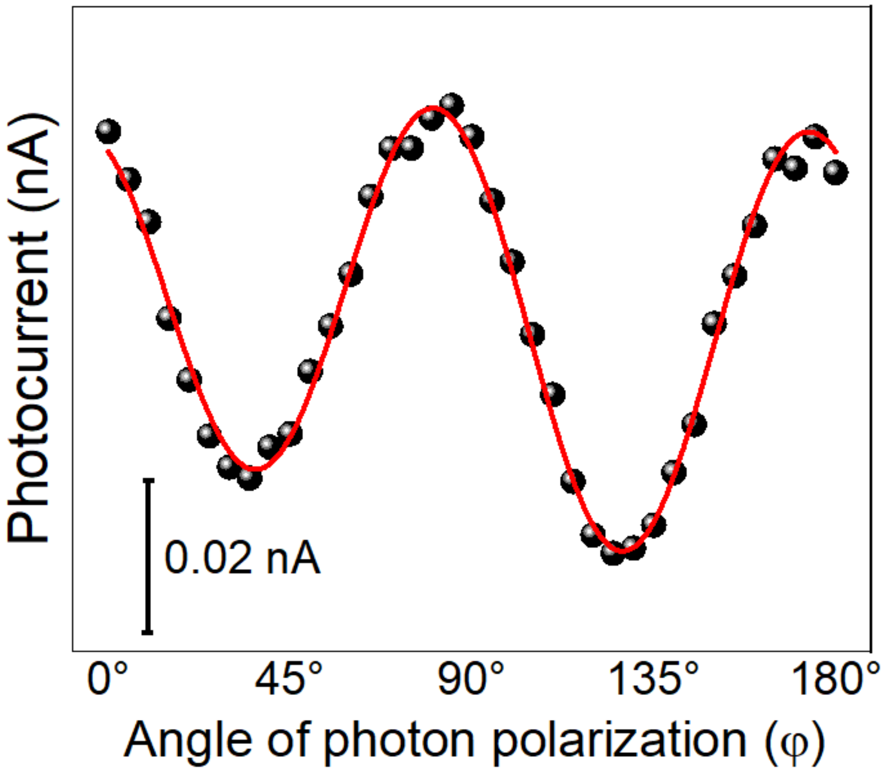

Figure S3 Angle of photon polarization dependence of photocurrent at an angle of incidence θ = 50° and at a fixed azimuthal angle of ζ = 180°.

Table S1 Extracted photocurrent contributions from the phenomenological photocurrent formula (Equation (4) in main text) fitting for various angle of incidence θ at a fixed azimuthal angle of ζ = 180°

θ (°)	C (pA)	L_1 (pA)	L_2 (pA)
30	-0.712	-4.81	81.62
40	3.95	-8.63	128
50	15.028	-13.26	203.42
60	19.28	-29.61	131.37
